# Evaluation of right atrial function by two-dimensional echocardiography and strain imaging in patients with RCA CTO recanalization

**DOI:** 10.1186/s12872-023-03108-y

**Published:** 2023-02-12

**Authors:** Recha Blessing, Ioannis Drosos, Thomas Münzel, Philip Wenzel, Tommaso Gori, Zisis Dimitriadis

**Affiliations:** 1 University Medical Center Mainz - Center of Cardiology, Johannes Gutenberg University, Mainz, Germany; 2 German Center for Cardiovascular Research (DZHK), Mainz Partner Site Rhine-Main, Mainz, Germany; 3 Division of Cardiology, Department of Medicine III, University Hospital Frankfurt, Goethe University Frankfurt am Main, Frankfurt, Germany; 4 Center for Thrombosis and Hemostasis (CTH), Johannes Gutenberg University, Mainz, Germany; 5 Department of Cardiology, University Medical Center Mainz, Langenbeckstr.1, 55131 Mainz, Germany; 6 Department of Cardiology, Center of Internal Medicine, Goethe University Frankfurt, Theodor-Stern-Kai 7, 60590 Frankfurt am Main, Germany

**Keywords:** Chronic total occlusion (CTO), Percutaneous coronary intervention (PCI), Coronary artery disease, Right atrium (RA), Two-dimensional speckle tracking echocardiography (2DE STE)

## Abstract

**Objectives:**

The right heart is mainly supplied with blood by the right coronary artery (RCA). The impact of RCA chronic total occlusion (CTO) on the function of the right heart [right atrium (RA) and ventricle (RV)] and whether successful recanalization of a RCA CTO improves the function of the right heart is not clearly understood yet. We aimed to evaluate right atrial function after recanalization of the RCA using transthoracic echocardiography with additional strain imaging.

**Methods and results:**

Fifty-five patients undergoing RCA CTO recanalization at the University Medical Center of Mainz were included in the study. Right atrial strain was assessed before and 6 months after successful CTO revascularization. The median age of the total collective was 66 (50–90) years. We did not find difference in our analysis of RA Volume (*p* 0.086), RA area (*p* 0.093), RA major dimension (*p* 0.32) and RA minor dimension (*p* 0.139) at baseline and follow-up. Mean RA reservoir strain at baseline was 30.9% (21.1–43.0) vs. 33.4% (20.7–47.7) at follow up (*p* < 0.001). Mean RA conduit strain was − 17.5% (− 10.7–(− 29.7)) at baseline vs. − 18.2% (− 9.6–(− 31.7)) at follow-up (*p* = 0.346). Mean RA contraction strain was − 12.9% (− 8.0- (− 21.3)) at baseline vs. − 15.5% (− 8.7–(− 26.6)) at follow-up (*p* < 0.001).

**Conclusion:**

Right atrial function was altered in patients with RCA CTO. Successful revascularisation of an RCA CTO improved RA function assessed by strain imaging at follow-up.

## Introduction

Coronary chronic total occlusions (CTO) are encountered in about 15–25% of patients with coronary artery disease undergoing coronary angiography. After initial neutral findings, recent evidence from clinical studies has demonstrated several benefits after successful CTO revascularization, especially in terms of angina relief and myocardial ischemia reduction, as well as regarding cardiac function [1]. The right and left atria play an important role in the maintenance and modulation of cardiac function. The right atrium (RA) modulates the filling of the right ventricle (RV) in three phases: reservoir, conduit and contraction. In the reservoir phase, the RA receives and stores blood from the venous system, as the tricuspid valve is closed. During diastole, the stored blood flows passively through the open tricuspid valve into the right ventricle, whereas the active filling of the right ventricle takes place in the contraction phase [2–5].

Studies demonstrated that the function of the RA is altered in patients with pulmonary hypertension, heart failure and amyloidosis and determines the prognosis of the patients [6–9]. An impaired right atrial function was also shown in patients with coronary artery disease and acute myocardial infarction [10–12]. The RA is routinely assessed by two-dimensional echocardiography (2DE). RA linear diameters, area and volume can be measured, but valid assessment of the RA by 2DE remains challenging [4, 13, 14]. In addition to the quantitative measurements of 2DE, two-dimensional (2D) speckle tracking echocardiography (2DE STE) allows the assessment of the RA function [15, 16]. Two-dimensional speckle tracking echocardiography is considered one of the best methods to assess atrial function [17–19].

In this study, we analysed RA function in reservoir, conduit and contraction phase in patients with chronic total occlusion of the right coronary artery using two-dimensional speckle tracking echocardiography and we evaluated whether successful recanalization of the CTO affects right atrial function.

## Materials and methods

### Study design and population

Patients who successfully underwent recanalization of RCA CTO at the University Medical Center in Mainz from July 2018 to September 2021 were enrolled in this prospectively conducted study after signing an informed consent.

**Fig. 1:**
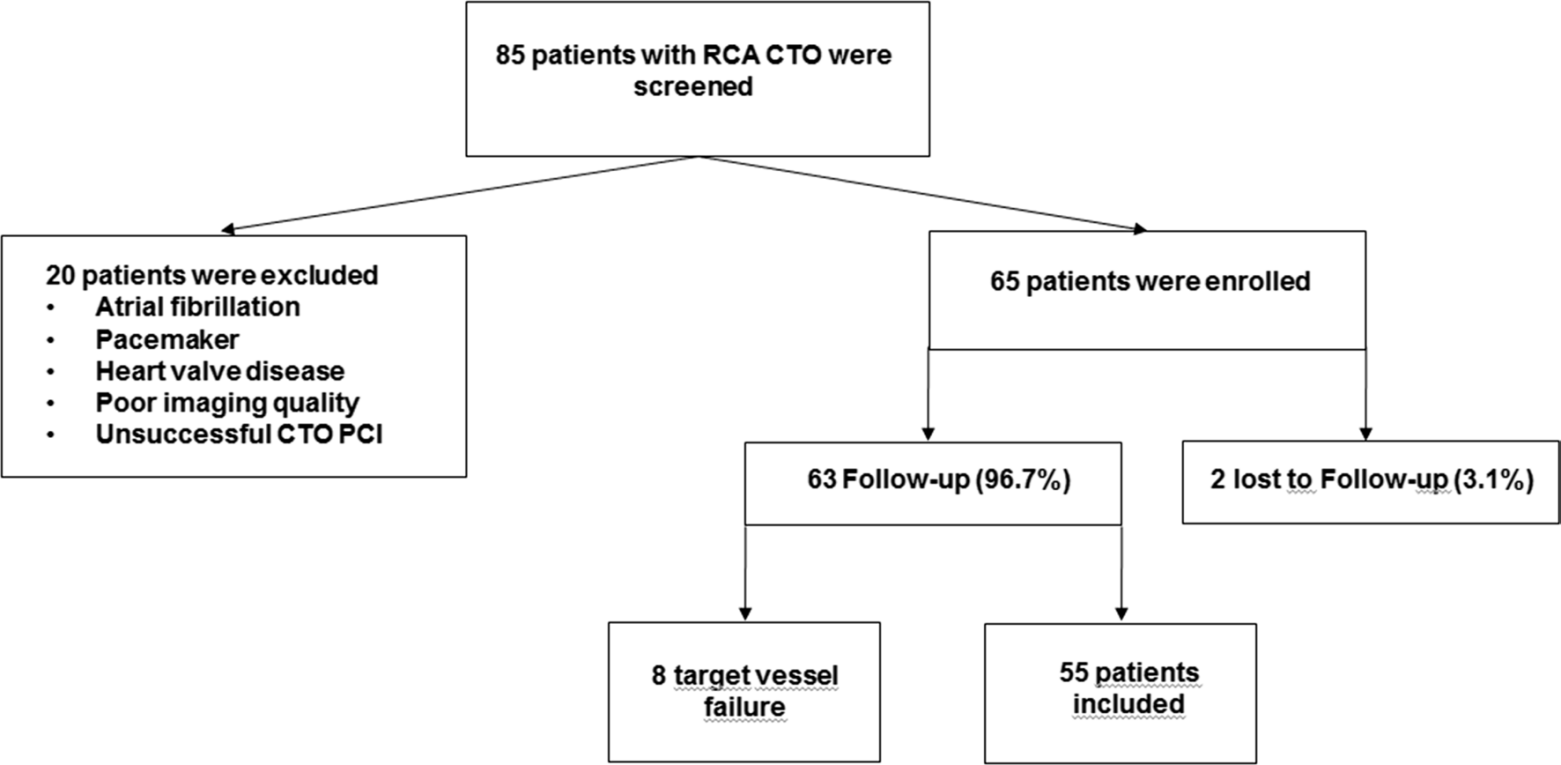
Flow-chart of enrollment

2DE and 2DE STE were performed at baseline (before RCA CTO PCI) and at 6 months follow-up. The study protocol was approved by the Ethics Committee of Rhineland Palatinate and was registered as a study in the DRKS. Patients with a target vessel failure (defined as presence of diameter restenosis > 50% by visual estimation, total re-occlusion or target vessel revascularization (defined as any revascularization within the treated vessel failure at 6 months follow-up surveillance coronary angiography) were excluded from the study (Fig. 1).

Inclusion criteria: proof of viability, successful recanalization of RCA CTO with good angiographic result in the 6 months surveillance coronary angiography, sinus rhythm and sufficient 2-dimensional imaging quality.

Exclusion criteria: valvular disease (moderate or severe valvular regurgitation or stenosis), valvular heart surgery or intervention, atrial fibrillation, paced rhythm, left or right bundle branch block, severe pulmonary, kidney (dialysis) or liver disease, heart surgery or intervention in the period between study inclusion and follow-up.

### Standard echocardiography and two-dimensional strain echocardiography

Conventional transthoracic echocardiography was performed at rest in a left lateral position using a Philips EPIQ 7 ultrasound system (Koninklijke Philips N.V., Amsterdam, Netherlands). Conventional echocardiographic assessment included two-dimensional views, including apical 4-chamber, apical 2-chamber and parasternal long-axis views and Doppler imaging analysis. Two cardiac cycles were recorded and analyzed in each view.

**Fig. 2:**
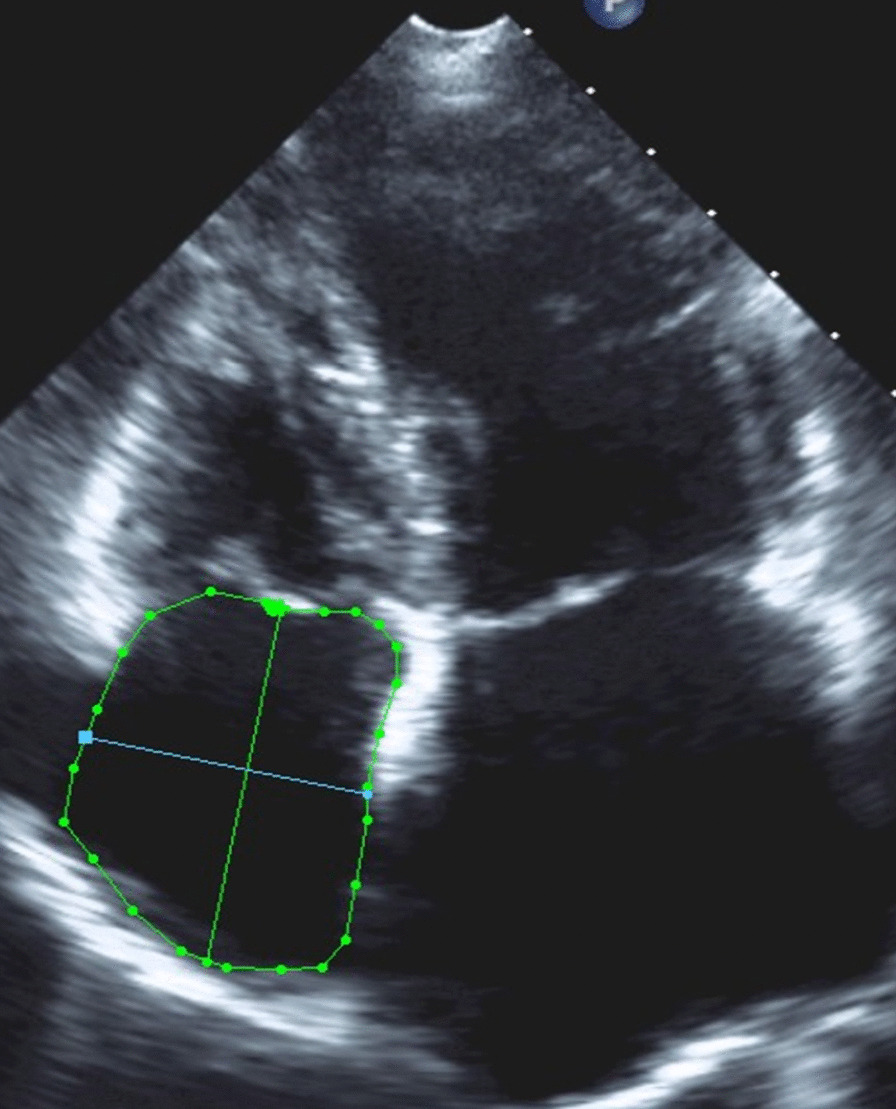
Representative example of RA assessment. *Blue line*: minor dimension, *green line*: major dimension, *green area*: RA area

For assessment of the right heart the RV-focused view was used in accordance to the guidelines of the American Society of Echocardiography [20]. RA area was measured by tracing the RA endocardium at the end of ventricular systole in the RV-focused four-chamber view. Right atrial volume RAVi (mL/m^2^) was indexed to the body surface area (BSA). The maximal long-axis distance was measured from the centre of the tricuspid annulus to the centre of the superior RA wall (major dimension), the minor distance was measured from the middle of the RA free wall to the interatrial septum (minor dimension) (Fig. 2) [13].

**Fig. 3:**
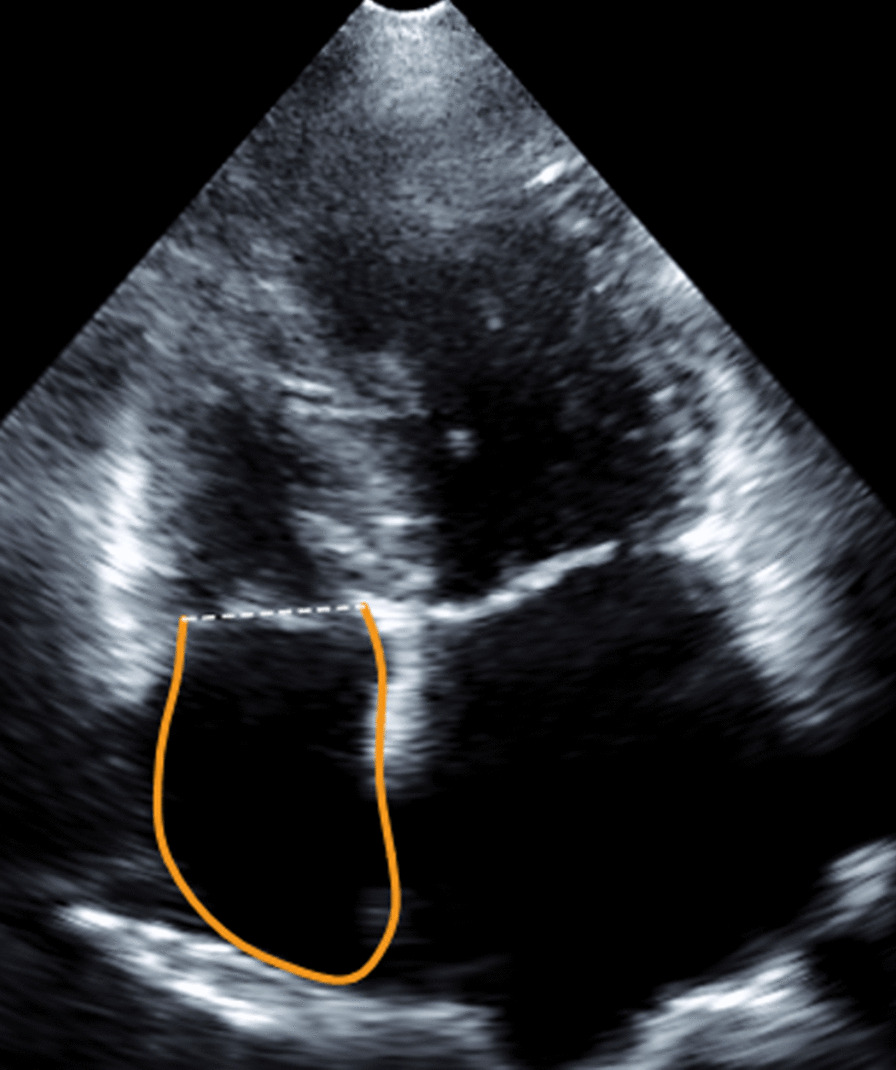
Representative example of RA strain imaging

The RA strain analysis was based on the consensus EACVI/ASE/Industry Task Force to standardize deformation imaging. For analysing the RA strain, the RV focused 4-chamber view was used, to visualize the entire RA and avoid RA shortening. The analysis was performed offline using Q-LAB 13 (PHILIPS Andover, MA Koninklijke Philips Electronics N.V. 2019). The region of interest (ROI) of the RA was traced along the endocardial border as follows: tricuspid valve annulus, RA lateral wall, RA roof, RA septal wall, ending at the opposite tricuspid annulus (Fig. 3) [16].

**Fig. 4:**
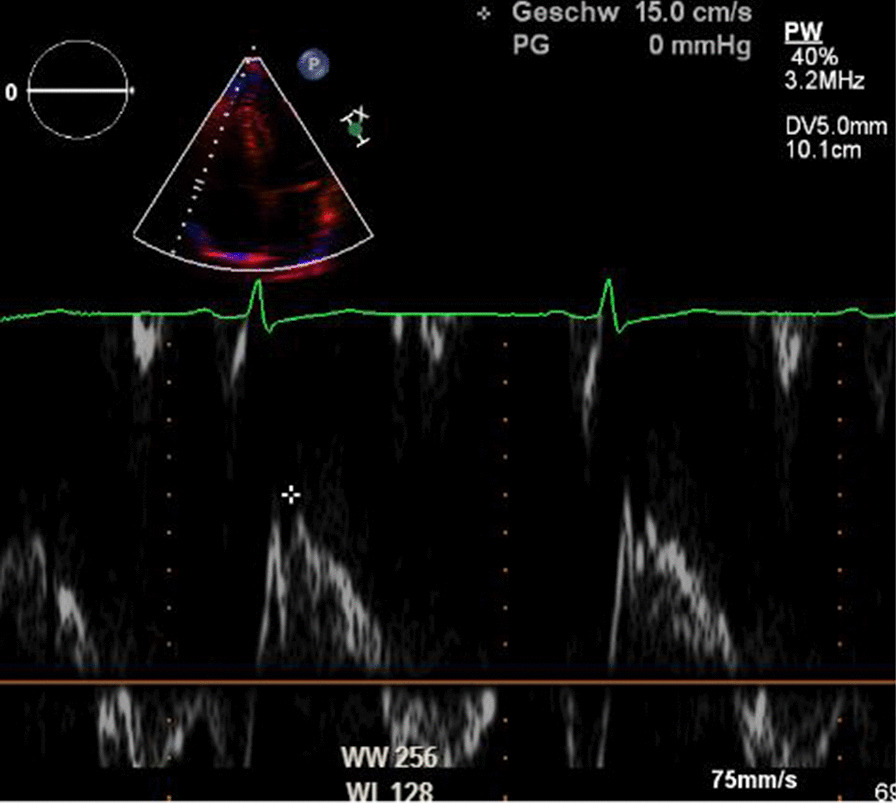
Representative example of RA assessment. TDI S’

**Fig. 5:**
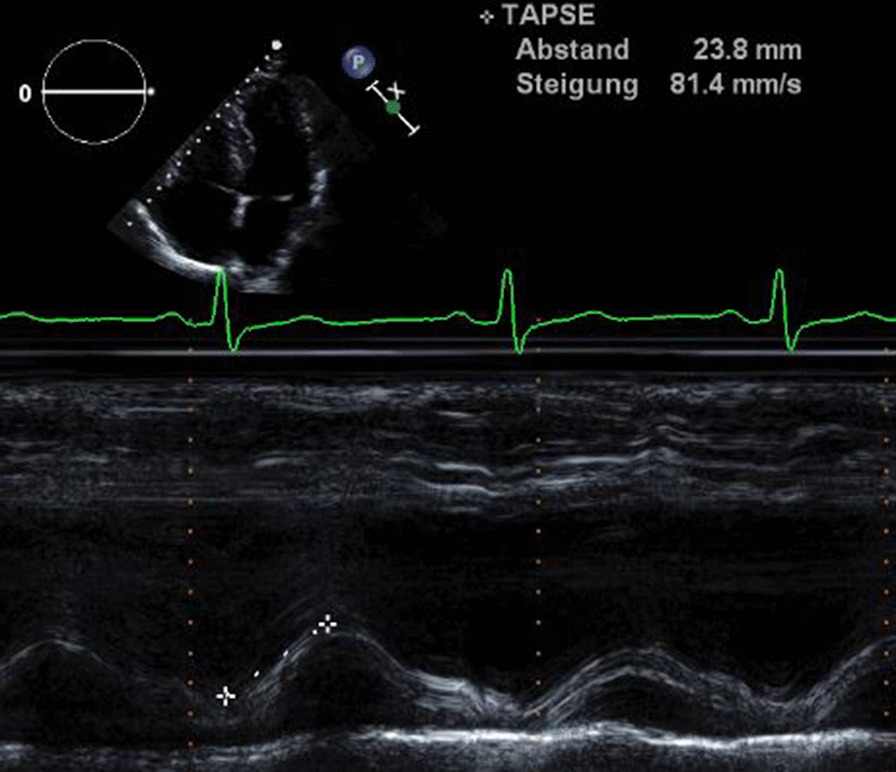
Representative example of RA assessment. TAPSE

RV systolic function was evaluated by TAPSE (tricuspid annular plane systolic excursion), RV FAC (fractional area change) and TDI S’ (Doppler–derived tricuspid lateral annular systolic velocity) in accordance to the guidelines of the American Society of Echocardiography. TAPSE is a parameter of RV longitudinal function and is measured from the tricuspid lateral annulus. FAC provides an index of the RV systolic function. RV was traced along the endocardial border including the apex and the lateral wall in both systole and diastole. TDI S’ is the systolic velocity of lateral tricuspid annulus by pulsed tissue Doppler) (Figs. 4,5) [13].

### Statistical analysis

Normal distribution of the variables was tested by QQ-plot analysis and the Kolmogorov–Smirnov test. Categorical data are presented as frequency and percentage, normally distributed data as mean ± standard deviation and not normally distributed variables are presented as median (minimum and maximum values). To compare means the Student`s t-test was used and medians the Mann–Whitney U-Test was performed. A two-sided *p* value of < 0.05 was considered to be statistically significant. The statistical analyses were performed using SPSS (Version 23, IBM SPSS Statistics).

## Results

### Clinical parameters

The data of 55 patients were analysed. All patients were in sinus rhythm at the time of the echocardiographic examination and showed no signs of (moderate or severe) valvular heart disease. Demographic parameters at baseline are shown in Table 1.

**Table 1:** Demographic characteristics at baseline

	All patients (n = 55)
Demographics characteristics	
Age, yrs	66 (50–90)
Male	39 (70.9%)
BMI (kg/m^2^)	26.6 (20–39.7)
Diabetes mellitus	12 (21.8)
Hypertension	51 (92.7)
Hyperlipidemia	48 (87.3)
Current smoking	18 (32.7)
Multivessel CAD	44 (78.6)
GFR (ml/min)	82 (35–117)
PAD	7 (12.7)
Previous MI	13 (23.6)
Previous PCI	36 (65.5)

Values represent n (%), median (minimum–maximum), or mean ± SD

yrs, years; BMI, body mass index; CAD, coronary artery disease; GFR, glomerular filtration rate; PAD, peripheral artery disease; MI = myocardial infarction; PCI, percutaneous coronary intervention

Fifty one (92.7%) of the patients had arterial hypertension and forty-eight (87.3%) had hyperlipidaemia. In addition, 70.9% of the collective were male, and the median age was 66 (50–90) years. Mean follow-up period was 192.5 ± 39 days.

Clinical parameters at baseline are shown in Table 2. When comparing the BNP values at baseline 66 pg/ml (10–788) and follow-up 66 pg/ml (10–892) we found no difference (*p* 0.88).

**Table 2:** Clinical parameters baseline and follow-up

	Baseline (n = 55)	Follow-Up (n = 55)	*p* value
NYHA			< 0.001
1	6 (10.9%)	35 (63.6%)	
2	37 (67.3%)	18 (32.7%)	
3	12 (21.8%)	2 (3.6%)	
CCS			< 0.001
0	9 (16.4)	41 (74.5%)	
1	14 (25.5)	5 (9.1%)	
2	22 (40)	9 (16.4%)	
3	10 (18.2)	0	
BNP	66 (10–788)	66 (10–892)	0.88

Values are median (minimum–maximum)

NYHA, New York Heart Association stages; CCS, Canadian Cardiovascular Society Classification, BNP, brain natriuretic peptide pg/ml

To assess the clinical value of a successful RCA CTO PCI we investigated the NYHA stages (New York Heart Association stages) and the CCS stages (CCS = Canadian Cardiovascular Society Classification) at baseline and follow-up. We found an improvement in NYHA and CCS stage in 34 patients ( 61.8%, *p* < 0.001) respectively in 38 patients ( 69.1%, *p* < 0.001). Complete freedom of angina was achieved in 74.5% of the patients in our collective and 63.6% of the patients reported to have no limitation of physical activity in daily life (NYHA stage 1) at follow-up (Table 2).

### Echocardiographic parameters

We found grade 1 diastolic dysfunction in 27 (49.1%) and grade 2 diastolic dysfunction in 8 (14.5%) patients. There was no evidence of diastolic dysfunction in 20 (36.4%) patients. At baseline mean LVEF (left ventricular ejection fraction) was 55% (20–59) and GLS (global longitudinal strain) was − 16% (− 6.1–(− 22.7)) and at follow-up mean LVEF was 55% (21–55) and GLS was − 16.2% (− 5.2–(− 25.6)). There was no significant difference of this values at baseline and follow-up (LVEF *p* 0.48 and GLS *p* 0.33). Compared to the values of the left ventricle (LVEF and GLS), we found significant differences of the values of the function of the right ventricle (TDI S, TAPSE and FAC). Results of the assessment of the right and left ventricular function is shown in Table 3.

**Table 3:** Left and right ventricular assessment

	Baseline (n = 55)	Follow-up (n = 55)	*p* value
Ventricular function			
TDI S’ (cm/sec)	12.30 ± 2.38	13.38 ± 2.62	< 0.001
TAPSE (mm)	20 (10–28)	24 (12–33.2)	< 0.001
FAC (%)	32.10 (14.06–45.03)	34.97 (16.71–44.17)	0.019
GLS (%)	− 16.0 (− 6.1–(− 22.7))	− 16.2 (− 5.2–(− 25.6))	0.33
LVEF (%)	55 (20–59)	55 (21–55)	0.48

Values are mean ± SD and median (minimum–maximum)

TAPSE, Tricuspid anular plane systolic excursion; FAC, fractional area change; GLS, global longitudinal strain; LVEF, Left ventricular ejection fraction; mm, millimetre; cm, centimetres; sec, seconds

Mean RA area was 16.65 ± 4.92 cm^2^ and mean RA volume was 43.89 ± 13.70 ml at baseline and 17.57 ± 5.86 cm^2^ and 44.83 ± 12.32 ml, respectively, at follow-up. The difference of baseline and follow-up values did not reach statistical significance (*p* 0.093 and *p* 0.086 for area and volume, respectively).

Analysis of the minor dimension at baseline vs. follow-up (39.29 ± 4.50 mm vs. 40.55 ± 5.85 mm, *p* 0.139) respectively the major dimension showed no difference (46.25 $\pm$ 6.36 vs. 46.87 ± 7.69 *p* 0.32). Right atrium dimensions and volumes were not altered compared to the reference ranges of the guidelines for the Echocardiographic Assessment of the Right Heart in Adults (2010) and guidelines of the European Association of Cardiovascular Imaging (2017) (RA area (reference: < 18 cm^2^ vs. 16.6 cm^2^), RA volume indexed for BSA (reference < 30 ml/m^2^ vs. 22.23 ml/m^2^), the RA minor dimension (reference < 4.4 cm vs. 3.9 cm) and RA major dimension (reference < 5.3 cm vs. 4.6 cm)). In the follow-up, values were still within the normal range [13, 14]. The results are presented in Table 4.

**Table 4:** RA quantitative assessment

	Baseline (n = 55)	Follow-up (n = 55)	*p* value
RA function			
RA minor dimension (mm)	39.29 ± 4.50	40.55 ± 5.85	0.139
RA major dimension(mm)	46.25 $\pm$ 6.36	46.87 ± 7.69	0.32
RA area (cm^2^)	16.65 ± 4.92	17.57 ± 5.86	0.093
RA volume (ml)	43.89 ± 13.70	44.83 ± 12.32	0.086
RAVi (mL/m2)	22.23 ± 6.58	22.75 ± 6.01	0.081

Values are mean ± SD

RA, right atrium; mm, millimetre; cm, centimetres; ml, millilitres; m, metres

Speckle tracking analysis of RA before RCA CTO PCI revealed following values: 30.9% (21.1–43.0) for RA reservoir strain, − 17.5% (− 10.7–(− 29.7)) for RA conduit strain and − 12.9% (− 8.0–(− 21.3)) for RA contraction strain. Comparing our values for the right atrial function assessed by two-dimensional speckle tracking echocardiography with the reference values recommended by the World Alliance of Societies of Echocardiography Study, we found an impaired right atrial reservoir and contraction strain function in patients with RCA CTO (RA reservoir strain reference: 45.8 ± 13% vs. 30.9 (21.2–43.0)%; RA contraction strain reference − 27.6 ± 9.7% vs. − 12.9 (− 8.0–(− 21.3)) [16]. The values of the conduit strain were within the normal range (reference − 18.4% ± 7.5 vs. − 17.5 (− 10.7–(− 29.7)) [21].

At follow-up RA reservoir strain was 33.4% (20.7–(− 47.7)), conduit strain − 18.2% (− 9.6–(− 31.7)) and contraction strain was − 15.5% (− 8.7–(− 26.6). Analysis showed a significant increase in the average values of RA reservoir and contraction strain.

**Fig. 6:**
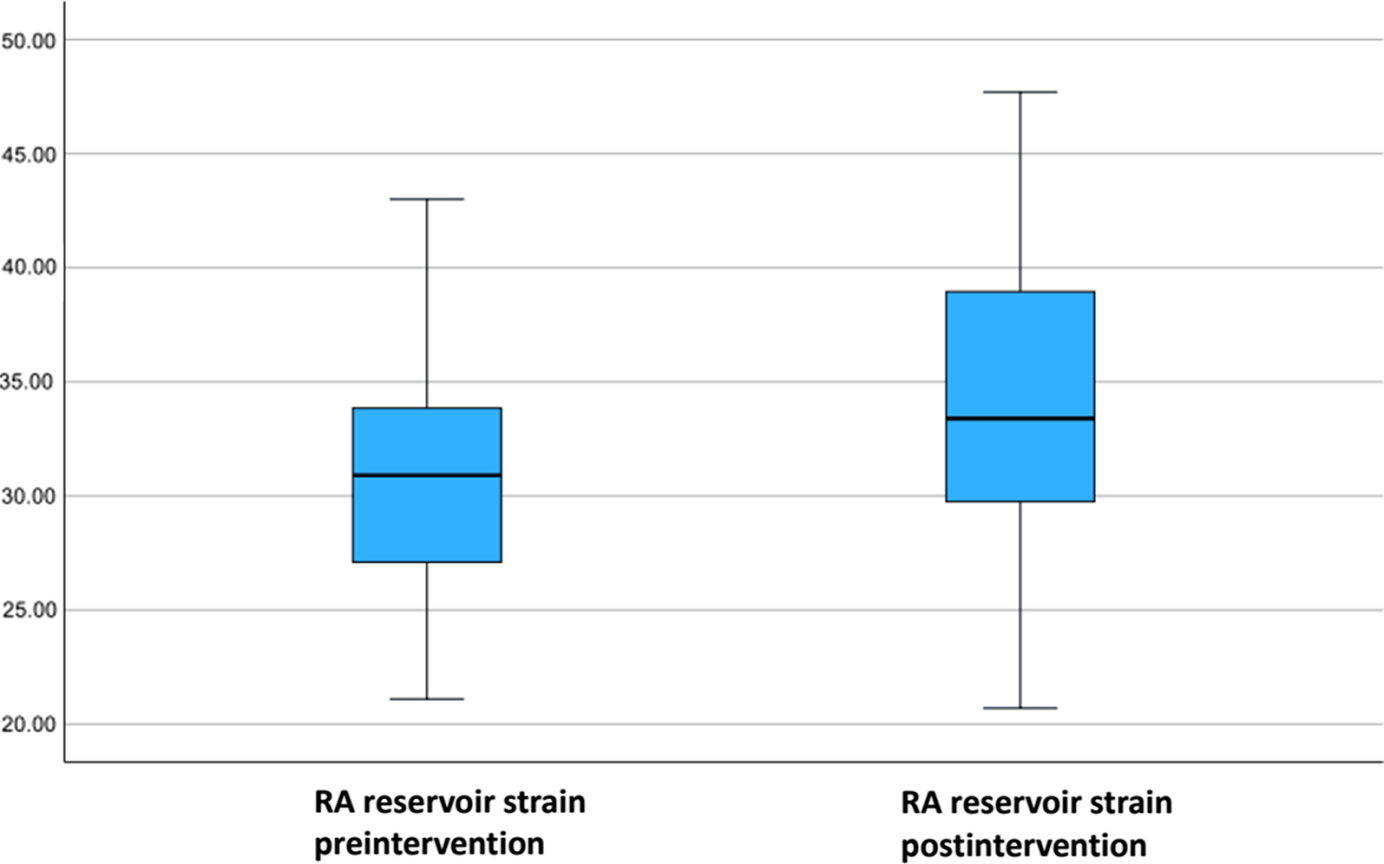
Boxplot diagram with RA reservoir strain at baseline before RCA CTO PCI and 6 months after successful RCA CTO PCI

**Fig. 7:**
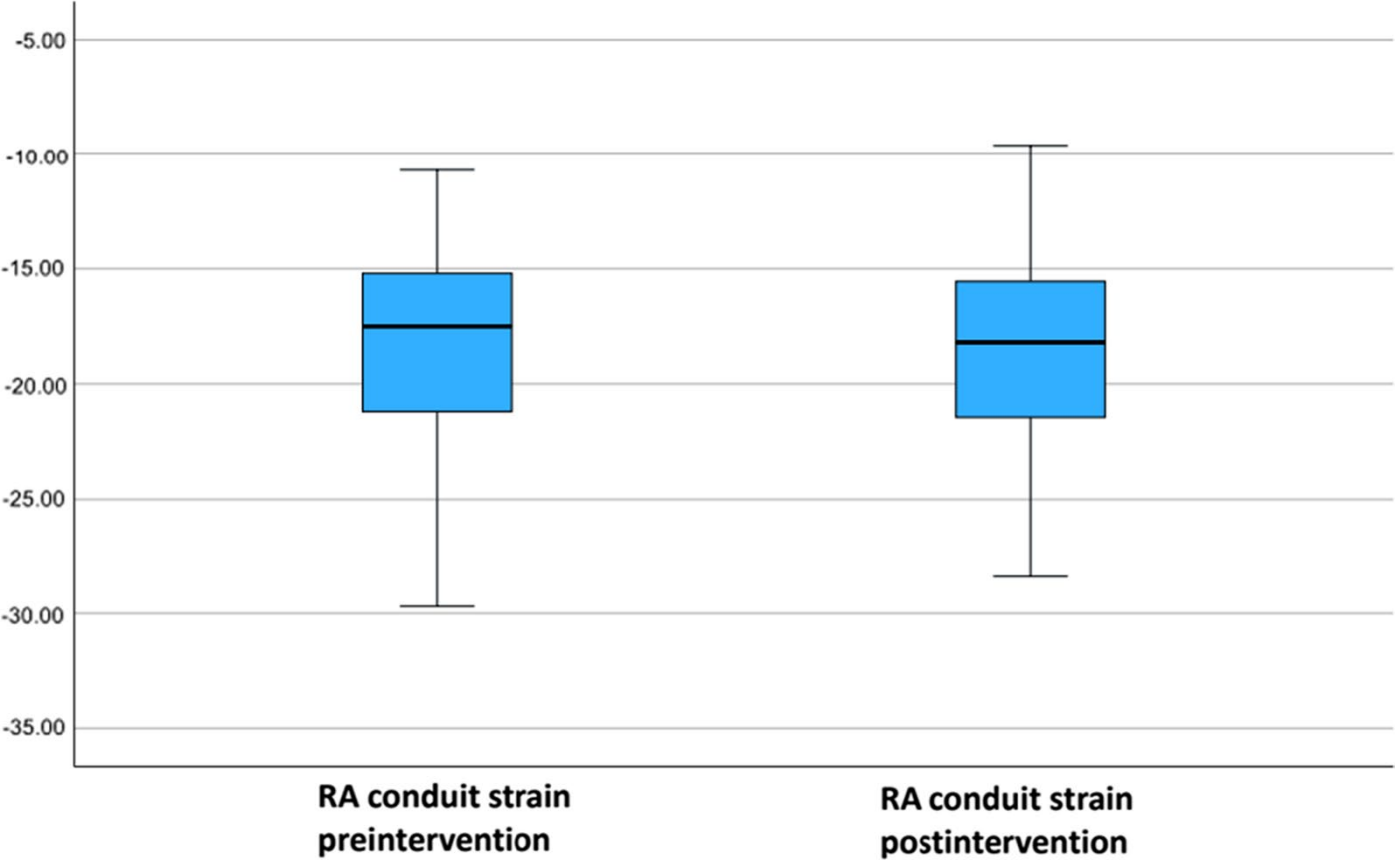
Boxplot diagram with RA conduit strain at baseline before RCA CTO PCI and 6 months after successful RCA CTO PCI

**Fig. 8:**
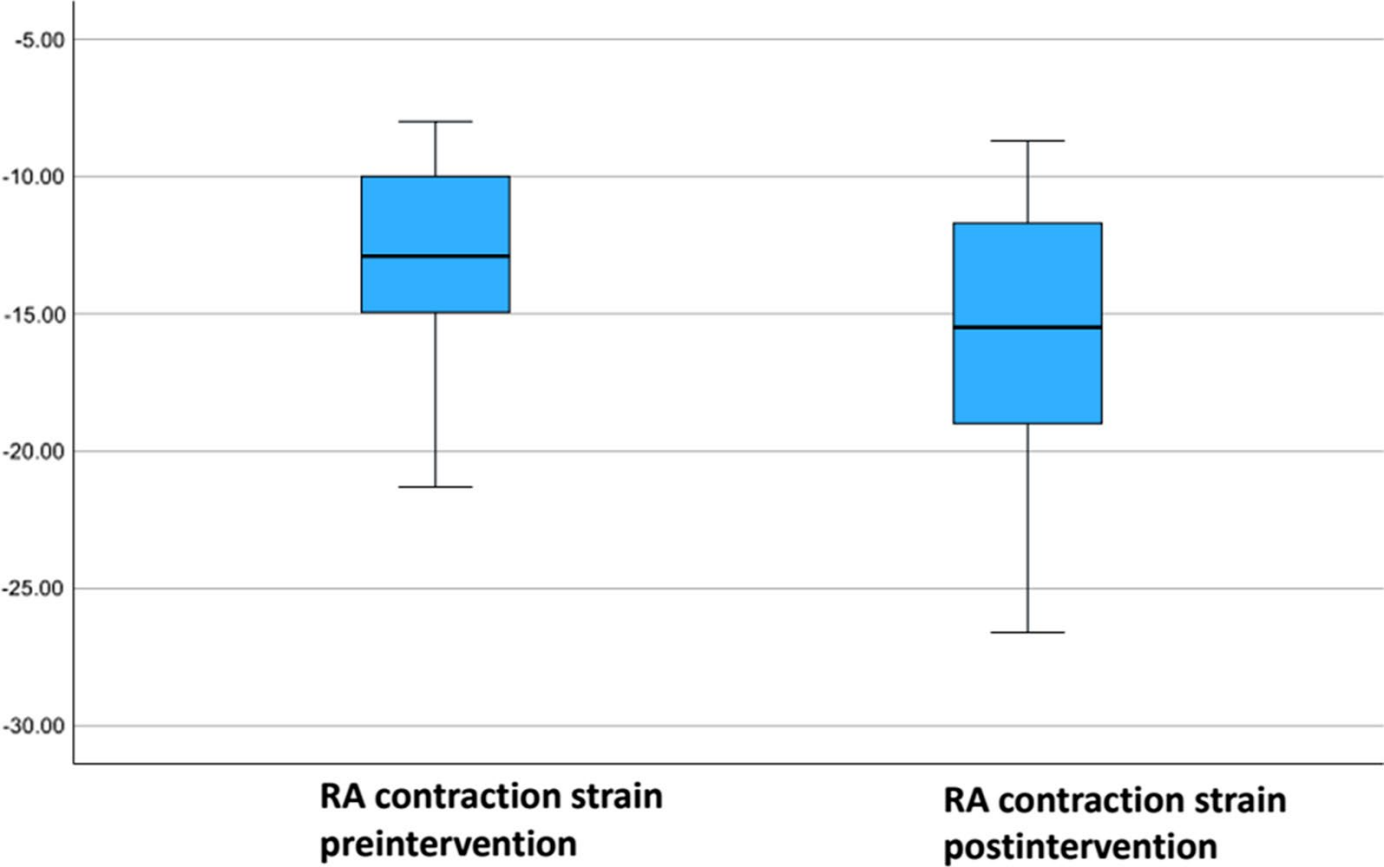
Boxplot diagram with RA contration strain at baseline before RCA CTO PCI and 6 months after successful RCA CTO PCI

The results of the assessment of the right atrial function are presented in Table 5 and Figs. 6, 7 and 8 (boxplot diagrams).

**Table 5:** RA function

	Baseline (n = 55)	Follow-up (n = 55)	*p* value
RA function			
RA reservoir strain (%)	30.9 (21.1–43.0)	33.4 (20.7− (− 47.7))	< 0.001
RA conduit strain (%)	− 17.5 (− 10.7–(− 29.7))	− 18.2 (− 9.6–(− 31.7))	0.346
RA contraction strain (%)	− 12.9 (− 8.0–(− 21.3))	− 15.5 (− 8.7–(− 26.6))	< 0.001

Values are median (minimum–maximum)

RA, right atrium

### Reproducibility

Inter-observer reproducibility was tested at baseline. Inter-observer reproducibility was good (intraclass correlation coefficient for reservoir strain: 0.84, 95% CI, 0.52–0.95, conduit strain 0.77, 95% CI, 0.32–0.92 and contraction strain 0.80 (0.41–0.93) as determined by intraclass correlation coefficients.

## Discussion

This study evaluated right atrial function in patients with RCA CTO and compared baseline two-dimensional speckle tracking echocardiography parameters with control parameters 6 months after successful recanalization. The major findings of the study include the following: (1) right atrial function assessed by 2DE STE is impaired in patients with chronic total occlusion of the right coronary artery compared to reference values; (2) successful recanalization increases reservoir and contraction strain, atrial dimension and volume is not altered; (3) health status of patients with successful RCA CTO PCI improved.

To date, studies described an improvement of the health status of patients after successful CTO PCI. Werner et al. found an improvement of angina frequency, quality of life and physical limitation in patients after successful CTO PCI compared to patients treated with optimal medical therapy at 12 month follow-up [30]. These findings are in good agreement with our results. The health status of the patients in our collective increased after successful CTO PCI, which could be shown by a change of NYHA and CCS stage. In CTO supplied myocardium a persistent ischemic zone and inducible ischemia was found [28, 31]. One explanation for the improvement of health status could be the improved perfusion and reduction of the ischemia of the myocardium supplied by the CTO vessel after PCI.

In contrast to the echocardiographic assessment of the right and left ventricle and the left atrium, the assessment of the right atrium is not yet part of the clinical routine. Standardized values of right atrial size and function were recently presented by the World Alliance of Societies of Echocardiography [21]. Before this recommendation, there was only limited data on reference values for the right atrial function, derived from studies in small populations [22–24].

Khedr et al. demonstrated in a collective with patients with stable CAD that the right atrial function is altered, atrial volumes and diameters were not affected. An altered right atrial function appears early in patients with stable CAD [10]. Eisvand et al. also found decreased right atrial function in patients with acute anterior myocardial infarction. In this collective RA contraction function was predictive of all-cause mortality or reinfarction during the follow-up period [25]. In a collective of 1235 patients with acute myocardial infarction (NSTEMI/STEMI) who underwent cardiac magnetic resonance imaging after successful PCI Schuster et al. found that the RA reservoir and the RA conduit function was impaired and predictive for onset of heart failure and MACE [12]. Our results are in good agreement with the findings above.

We showed an impaired right atrial reservoir and contraction strain function assessed by two-dimensional speckle tracking echocardiography in our collective. In a collective of 70 consecutive patients with inferior myocardial infarction and right ventricular infarction Nourian et al. showed an impaired atrial function assessed by 2DSTE. One reason could be the impaired right ventricular function in patients with right ventricular infarction, which affects the function of the right atrium [11].

In our collective of patients with RCA CTO we found decreased right atrial and right ventricular function. Since ventricular function strongly influences atrial function, this could be an explanation for the decreased phasic RA function and its improvement after successful recanalization as the right heart is mainly supplied with blood by the right coronary artery [26]. In CTO-supplied myocardium ischemia and impaired microvascular function occur. Microvascular function and blood circulation improves after successful revascularization of a CTO vessel [27–29] and improved perfusion of the myocardium results in better function of the right heart and health status of the patients in our collective.

### Study limitations

Apart from coronary artery disease, RA function could have been altered due to other factors (e.g. high blood pressure, obesity or gender). Also, RA strain imaging is not yet part of the clinical routine*.* Due to the small sample size and our hypothesis (that successful recanalization of RCA CTO affects RA function), no regression analysis could be performed. Our study is hypothesis generating, studies with larger collectives are needed to confirm our results and show if altered right atrial function has a prognostic value in the CTO PCI setting.

## Conclusion

In our study, we found that right atrial reservoir and contraction strain was impaired in patients with a chronic total occlusion of the right coronary artery. We demonstrated that right atrial reservoir and contraction strain was improved after successful recanalization, whereas conduit strain remained unaffected.

## Data Availability

The dataset generated and/or analysed during the current study are available from the corresponding author on reasonable request.

## Abbreviations

2DE: Two-dimensional echocardiography
2DE STE: Two-dimensional speckle tracking echocardiography
BMI: Body mass index
BSA: Body surface area
CAD: Coronary artery disease
CCS: Canadian Cardiovascular Society classification
cm: Centimetre
CTO: Coronary chronic total occlusions
FAC: Fractional area change
GFR: Glomerular filtration rate
GLS: Global longitudinal strain
LVEF: Left ventricular ejection fraction:
MACE: Major adverse cardiac event
MI: Myocardial infraction
ml: Millilitre
mm: Millimetre:
NSTEMI: Non-ST-segment elevation myocardial infarction
NYHA: New York Heart Association classification
RA: Right atrium
RAVi: Right atrial volume
RCA: Right coronary artery (RCA)
ROI: Region of interest
RV: Right ventricle
PAD: Peripheral artery disease
PCI: Percutaneous coronary intervention
STEMI: Non-ST-segment elevation myocardial infarction
TAPSE: Tricuspid annular plane systolic excursion
TDI S’: Doppler–derived tricuspid lateral annular systolic velocity
Yrs: Years
